# Adverse Birth Outcomes and Maternal Exposure to Trichloroethylene and Tetrachloroethylene through Soil Vapor Intrusion in New York State

**DOI:** 10.1289/ehp.1103884

**Published:** 2011-12-05

**Authors:** Steven P. Forand, Elizabeth L. Lewis-Michl, Marta I. Gomez

**Affiliations:** Bureau of Environmental and Occupational Epidemiology, New York State Department of Health, Troy, New York, USA

**Keywords:** birth defects, birth outcomes, soil vapor intrusion, tetrachloroethylene, trichloroethylene, volatile organic compounds

## Abstract

Background: Industrial spills of volatile organic compounds (VOCs) in Endicott, New York (USA), have led to contamination of groundwater, soil, and soil gas. Previous studies have reported an increase in adverse birth outcomes among women exposed to VOCs in drinking water.

Objective: We investigated the prevalence of adverse birth outcomes among mothers exposed to trichloroethylene (TCE) and tetrachloroethylene [or perchloroethylene (PCE)] in indoor air contaminated through soil vapor intrusion.

Methods: We examined low birth weight (LBW), preterm birth, fetal growth restriction, and birth defects among births to women in Endicott who were exposed to VOCs, compared with births statewide. We used Poisson regression to analyze births and malformations to estimate the association between maternal exposure to VOCs adjusting for sex, mother’s age, race, education, parity, and prenatal care. Two exposure areas were identified based on environmental sampling data: one area was primarily contaminated with TCE, and the other with PCE.

Results: In the TCE-contaminated area, adjusted rate ratios (RRs) were significantly elevated for LBW [RR = 1.36; 95% confidence interval (CI): 1.07, 1.73; *n* = 76], small for gestational age (RR = 1.23; 95% CI: 1.03, 1.48; *n* = 117), term LBW (RR = 1.68; 95% CI: 1.20, 2.34; *n* = 37), cardiac defects (RR = 2.15; 95% CI: 1.27, 3.62; *n* = 15), and conotruncal defects (RR = 4.91; 95% CI: 1.58, 15.24; *n* = 3). In the PCE-contaminated area, RRs for cardiac defects (five births) were elevated but not significantly. Residual socioeconomic confounding may have contributed to elevations of LBW outcomes.

Conclusions: Maternal residence in both areas was associated with cardiac defects. Residence in the TCE area, but not the PCE area, was associated with LBW and fetal growth restriction.

After a 1979 spill of 4,100 gallons of 1,1,1-trichloroethane (TCA) at a large IBM semiconductor manufacturing facility in the Village of Endicott, New York, groundwater sampling conducted in 1980 revealed the presence of a mixture of contaminants beneath the site and nearby residential and commercial areas. Groundwater sampling identified a larger than expected plume of contaminants, including trichloroethylene (TCE) and tetrachloroethylene [perchloroethylene (PCE)], along with lesser amounts of TCA, dichloroethane, dichloroethene, methylene chloride, vinyl chloride, freon 113, and other volatile organic compounds (VOCs), with total groundwater VOC concentrations ranging from 5 to > 500 µg/L [New York State Department of Environmental Conservation (NYSDEC) 2003]. Although the TCA spill initiated the extensive groundwater sampling, the other contaminants discovered were the result of prior spills and releases. The contaminant plume extended from the 135-acre facility southward beneath approximately 70 blocks of the Village of Endicott, a community of 13,000 located in upstate New York. The groundwater has been treated and monitored since then.

Until recently, environmental scientists generally assumed that VOC-contaminated groundwater near hazardous waste sites resulted in potentially harmful exposures to VOCs only if the groundwater was used as a drinking water source. Because the residential drinking water in Endicott is supplied primarily from wells outside the plume area, it was assumed that residents were not being significantly exposed to contaminants from the plume. Beginning in the late 1990s, new information emerged showing that contaminants volatilized and moved through air pockets in soil into structures above or near contaminant plumes through a process known as soil vapor intrusion (SVI) [New York State Department of Health (NYSDOH) 2006].

In 2000–2001, concerns about SVI in Endicott led to expanded off-site soil vapor sampling. TCE was the predominant contaminant in soil vapor, with relatively small fractions of TCA and PCE. Levels of TCE in soil vapor above the plume typically ranged from 100 to 10,000 µg/m^3^ (McDonald and Wertz 2007; NYSDEC 2003). Data from all indoor air sampling showed TCE levels ranging from 0.18 to 140 µg/m^3^. In the area with the highest off-site contaminant levels (the core area), the median indoor air level of TCE was 16 µg/m^3^ (NYSDEC 2003; Sanborn, Head & Associates 2005). Data compiled from 15 U.S. indoor air studies that measured background concentrations of VOCs in homes not expected to be influenced by SVI show median background indoor air concentrations for TCE ranging from the reference limit to 1.1 µg/m^3^, with the 95th percentile ranging from 0.6 to 3.3 µg/m^3^ [U.S. Environmental Protection Agency (EPA) 2011]; 81% of the indoor air levels in the core area in Endicott were > 0.6 µg/m^3^, and 67% were > 3.3 µg/m^3^, the lower and upper 95th percentile range values (NYSDEC 2003). A second, smaller area of contamination was also found where PCE predominated. PCE levels in this area ranged from 0.1 to 24 µg/m^3^ in indoor air, whereas median background U.S. indoor air concentrations for PCE have ranged from the reference limit to 2.2 µg/m^3^, and the 95th percentile ranged from 4.1 to 9.5 µg/m^3^ (U.S. EPA 2011). Duration of exposures before the discovery of SVI is unknown, but exposures through this pathway may date back at least to the 1970s. To reduce exposures, mitigation systems were installed in > 450 structures beginning in 2002.

Although there are numerous studies on the carcinogenicity of TCE and PCE, relatively few studies have looked at reproductive and developmental health outcomes associated with these chemicals. Previous studies of women living in areas with TCE- or PCE-contaminated drinking water have suggested an association between TCE exposure, and cardiac defects, oral clefts, neural tube defects (NTDs), and choanal atresia [Bove et al. 1995; Goldberg et al. 1990; MDPH (Massachusetts Department of Public Health), CDC (Centers for Disease Control and Prevention), and MHRI (Massachusetts Health Research Institute)1996]. Studies of women exposed to PCE in drinking water have suggested increased prevalence of oral clefts and NTDs (Aschengrau et al. 2009; Bove et al. 1995). One study showed associations between potential exposures to TCE in ambient air and congenital heart defects in infants born to women ≥ 38 years of age (Yauck et al. 2004).

Studies of women exposed to TCE-contaminated drinking water have shown some evidence of increases in low birth weight (LBW) or very LBW, term LBW, and small for gestational age (SGA) births [Agency for Toxic Substances and Disease Registry (ATSDR) 1998; Bove et al. 1995; MDPH, CDC, and MHRI 1996; Rodenbeck et al. 2000]. A study in Camp LeJeune, North Carolina, found a modest association between PCE in drinking water and SGA (Sonnenfeld et al. 2001).

In this report, we present the findings from an exploratory community study of adverse birth outcomes among residents living in an area with SVI by TCE and PCE. The Endicott SVI exposure area is one of the earliest such sites to be identified and the largest site in New York State to receive this type of health outcome investigation.

## Materials and Methods

*Study design.* We compared birth outcomes among residents of the exposed community and birth outcomes in the general New York State population using multivariate Poisson regression analyses. Existing birth certificate and birth defect registry records were used to obtain information on outcomes and maternal and child risk factors at the individual level, and exposure was classified at the group level.

The study was reviewed and approved by the NYSDOH Institutional Review Board, which waived informed consent because the study was of no or minimal risk to the subjects and could not be practicably carried out without a waiver.

*Study population.* The study population included all live births in the Endicott study area, and the comparison population included all births in the rest of New York State, excluding New York City. LBW, preterm, and fetal growth restriction outcomes were studied for singleton births from 1978 to 2002; birth defects were studied for all births from 1983 to 2000. These years were chosen to most closely match the exposure years for which birth outcome data were available.

Birth certificate data from New York State Vital Statistics files (NYSDOH Vital Statistics Unit, Bureau of Biometrics and Health Statistics, Menands, NY) were used to examine LBW, preterm births, and fetal growth restriction among singleton births. The birth weight and preterm outcomes are defined as follows: LBW (< 2,500 g), very LBW (< 1,500 g), preterm birth (< 37 weeks gestation), and very preterm birth (< 32 weeks gestation). The growth restriction outcomes are term LBW (≥ 37 weeks gestation and birth weight < 2,500 g) and SGA. SGA is defined as a birth weight below the 10th percentile of the New York State (excluding New York City) birth weight distribution of singleton births stratified by gestational week, sex, and 5-year time period (1978–1982 through 1998–2002) (Alexander et al. 1996).

Birth records with missing birth weight or birth weight < 100 g or > 8,000 g were excluded from the birth weight analyses (0.3% of the birth records). Records missing gestational age or with gestational ages < 12 weeks or > 50 weeks were excluded from the preterm analyses (3.2% of the birth records). For the SGA and term LBW analyses, exclusions for implausible birth weight/gestational age combinations follow those of Alexander et al. (1996) (5.9% of the birth records). Plausible combinations by gestational week ranged from 125 to 1,250 g at 20–21 weeks to 1,000–6,000 g at ≥ 38 weeks. The percentage of birth records excluded because of missing information was similar for the study and comparison populations.

Birth defects diagnosed among infants up to 2 years of age for births were ascertained from the NYSDOH Congenital Malformations Registry (CMR) (Troy, NY). Specific birth defects were identified using codes from the *International Classification for Diseases, 9th Revision* (ICD-9) (U.S. Department of Health and Human Services 1991). The CMR linked the birth defect records to their corresponding birth certificate record to obtain information on residence at birth and maternal and infant characteristics. Defects were grouped into two broad categories: all reportable birth defects, and a subset of all reportable birth defects called “surveillance birth defects.” Reportable birth defects include all major birth defects required to be reported to the CMR (NYSDOH 2004). Surveillance birth defects, which account for about half of all reportable birth defects, include about 80 types of birth defects that are easily recognizable and consistently and accurately diagnosed by physicians (Holmes 1999). In addition, several specific subsets of defects showing associations with TCE and/or PCE in other studies were examined. These include all cardiac defects [ICD-9 codes 745.0–747.9; for infants with patent ductus arteriosus (747.0), only those ≥ 2,500 g at birth were included because this is not considered an inherent abnormality in premature infants], major cardiac defects (745.0, 745.1, 745.2, 746.0, 746.1, 746.3, 746.4, 746.7, 747.1, 747.3), and conotruncal heart defects (745.0, 745.1, 745.2). NTDs (740, 741, 742.0), orofacial clefts (749.00–749.04, 749.10–749.14, 749.20–749.25), and choanal atresia (748.00), a defect of the nasal airway developmentally similar to conotruncal heart defects, were also of interest because of previous association with TCE or PCE exposure.

**Figure 1:**
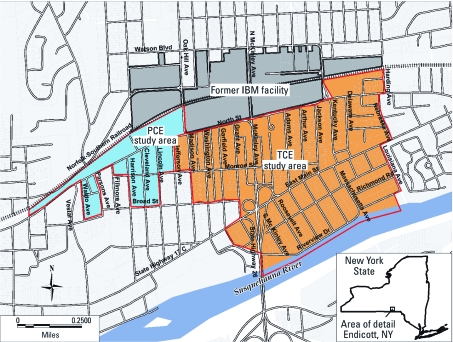
The TCE and PCE study areas and the location of the former IBM manufacturing facility, Village of Endicott, New York (USA).

*Exposure assessment.* Approximately 25% of structures in the area potentially affected by SVI were sampled for indoor air VOC levels. Structures near the periphery were oversampled because the sampling’s primary purpose was to identify a boundary beyond which there were no discernible impacts on indoor air, for mitigation purposes. For this reason, we could not assign exposures at the household level or assign exposure gradients. Because of differences in the predominant contaminants, two exposure areas were identified: a primary exposure area located south of the IBM facility (the TCE area) and a smaller, contiguous area, to the west (the PCE area) (Figure 1).

The TCE and PCE area boundaries were drawn using village block boundaries that generally captured the population of the area where mitigation systems were offered. This was accomplished by overlaying a digital map of the 2000 U.S. Census blocks (U.S. Census Bureau 2000) on the areas of contamination using a geographic information system (MapInfo, version 7.8; MapInfo Corp., Troy, NY). Because the census blocks in Endicott are relatively small, the study area boundaries closely coincided with the area where the inferred VOC presence in soil gas ranged from 10 µg/m^3^ to > 10,000 µg/m^3^ (NYSDEC 2003).

Exposure status was assigned at the group level based on residence within the TCE or PCE areas. We used the mother’s residential address at the time of birth to assign births to the study areas. Addresses were geocoded to a latitude and longitude using Mapmarker Plus (version 10.0; MapInfo Corp.). If an address was not automatically geocoded, it was manually assigned a latitude and longitude using tax parcel data (New York State Department of Taxation and Finance, Office of Real Property Tax Services, Albany, NY), NYS New York State digital street files (New York State Division of Homeland Security & Emergency Services, Office of Cyber Security, Albany, NY), ZIP+4 files (U.S. Postal Service, National Customer Support Center, Memphis, TN), and street-by-street ZIP code directories (U.S. Postal Service 1985, 2000). Thus, a birth was considered exposed if the mother resided in one of the study areas at the time of birth, and the general population of New York State (excluding New York City) residing outside of the study areas was considered unexposed.

*Statistical analysis.* We used Poisson regression to analyze the relationship between exposure status and adverse birth outcomes. Data on potential confounders (factors most frequently associated with birth outcomes) were obtained from the birth certificate records. The following covariates were included: mother’s age (< 19, 19–34, ≥ 35 years), education (less than high school, high school to some college, ≥ 4 years of college), race (white, nonwhite), infant’s sex, number of previous live births (0, 1, ≥ 2), and adequate prenatal care (based on the modified Kessner index, which uses start of prenatal care, number of prenatal visits, and duration of pregnancy to determine adequacy of prenatal care) (Kessner et al. 1973). Separate analyses were done for the PCE area, the TCE area, and both areas combined. We present the adjusted rate ratios (RRs) and 95% confidence intervals (CIs). An adjusted RR with a chi-square *p*-value < 0.05 was considered statistically significant. All analyses were performed using SAS (version 9.1; SAS Institute Inc., Cary, NC).

Because smoking is a major risk factor for several of these outcomes, we conducted a subanalysis of LBW and fetal growth restriction that included an additional term for smoking status (nonsmoker vs. < 0.5 pack, 0.5–1 pack, > 1 pack per day), as reported by the mother on the birth certificate. Although this information has been collected since 1988, we limited this subanalysis to 1998–2002 (5 years) because these smoking data are more complete.

## Results

Table 1 presents the demographic characteristics of the Endicott study areas and New York State (excluding New York City) for 1980 and 2000. The population of the TCE area accounts for about 80% of the population of the combined study area. Between 1980 and 2000, the population of the combined study area declined 15%, whereas the New York State population increased about 5%. The combined study area had a lower percentage of minorities than the rest of the state. Although the statewide percentage of households living below the poverty level remained steady from 1980 to 2000, at about 10%, it increased from 12% to 24% in the study area. In addition, the median household income in the study area fell from two-thirds of the statewide median in 1980 to half of the statewide median in 2000. The demographic characteristics of the TCE and PCE areas were similar (data not shown).

**Table 1:** Demographic characteristics of the Endicott, New York (USA), exposure area and New York State (NYS), excluding New York City (NYC), 1980 and 2000.

1980*^a^*			2000*^b^*		
Characteristic	Endicott exposure area	NYS excluding NYC	Endicott exposure area	NYS excluding NYC
Population (*n*)								
Total population (*n*)		3,540		10,486,433		3,002		10,968,179
TCE exposure area		2,851		—		2,378		—
PCE exposure area		689		—		624		—
Females of reproductive age (*n*)		765		2,497,016		686		2,297,231
Race and ethnicity (%)								
White		97		92		89		85
Black		1		6		5		8
Other		2		2		4		5
Multiracial		—		—		2		2
Percent Hispanic (%)		2		2		2		6
Percent minority (%)*c*		5		9		12		18
Economic characteristics								
Median household income		$12,668		$18,889		$23,421		$47,517
Persons below poverty level (%)		12		9		24		10
**a**U.S. Census Bureau (1981, 1982). **b**Data from U.S. Census Bureau (2001, 2002). **c**Includes Hispanics, African Americans, Asians, Pacific Islanders, and Native Americans.								

Geocoding identified 1,440 live births to mothers living within the combined study area during 1978–2002. There were 1,090 births in the TCE area and 350 births in the PCE area. The remaining 3.6 million births from 1978 through 2002 in New York State (excluding New York City) were assigned to the comparison population. Because of the small number of births in the study area, there were very few exposed cases of some of the rarer outcomes. This was more apparent in the PCE area, where only one exposed case each of conotruncal defects, very preterm births, and very LBW births were observed. Term LBW in the PCE area and several categories of heart defects in both areas had fewer than 10 exposed cases. The number of exposed cases of each outcome is given with the results in Tables 2 and 3. Results based on small numbers should be interpreted cautiously.

RR estimates for the covariates included in the models for LBW, SGA, term LBW, and surveillance, major cardiac, and conotruncal birth defects are shown in Table 4 for the TCE area. LBW, SGA, and term LBW were associated with maternal age > 35 years, lower education, nonwhite race, no previous births, inadequate prenatal care, and infant female sex. Surveillance defects and cardiac birth defects were associated with maternal age > 35 years, lower education, and infant male sex. In addition, cardiac defects were associated with nonwhite race and previous live births; however, these factors were inversely associated with surveillance birth defects. Adequate prenatal care was inversely associated with major cardiac defects.

The results from the Poisson regressions of birth weight, prematurity, and growth restriction outcomes are presented in Table 2. In the PCE area, none of the adjusted RRs were statistically significant, and all had RRs < 1.0 with the exception of SGA, which had an RR of 1.04. Very LBW, term LBW, and very preterm all had fewer than five cases, as noted above; thus, these results should be interpreted with caution. In the TCE area, significantly elevated RRs were seen for LBW (RR = 1.36; 95% CI: 1.07, 1.73; *n* = 76), SGA (RR = 1.23; 95% CI: 1.03, 1.48; *n* = 117), and term LBW (RR = 1.68; 95% CI: 1.20, 2.34; *n* = 37). Very LBW was also elevated in the TCE area, although not significantly so (RR = 1.61; 95% CI: 0.94, 2.78; *n* = 14). In the combined study area the RRs were significantly elevated for SGA and term LBW, a reflection of the results for the TCE area.

During 1998–2002, 39% of mothers in the study areas and 14% of mothers statewide reported smoking during the pregnancy. In the combined study area smoking was significantly associated with LBW (*n* = 22), SGA (*n* = 35), and term LBW (*n* = 11), and the association increased with the number of cigarettes smoked for each outcome (data not shown). With smoking status included in the models, RRs decreased for all outcomes: for LBW, from 1.60 (95% CI: 1.01, 2.54) to 1.37 (95% CI: 0.86, 2.18); for SGA, from 1.54 (95% CI: 1.09, 2.16) to 1.31 (95% CI: 0.93, 1.85); and for term LBW, from 2.56 (95% CI: 1.42, 4.62) to 2.08 (95% CI: 1.15, 3.76).

**Table 2:** Adjusted RRs*^a^* (95% CIs) for adverse birth outcomes in the PCE, TCE, and combined study areas, Village of Endicott, New York (USA), 1978–2002.

PCE area			TCE area			Combined study area		
Adverse birth outcome	*n*	RR (95% CI)	*n*	RR (95% CI)	*n*	RR (95% CI)
LBW		12		0.70 (0.39 , 1.27)		76		1.36 (1.07, 1.73)*		88		1.20 (0.96, 1.50)
Very LBW		1		0.39 (0.06, 2.78)		14		1.61 (0.94, 2.78)		15		1.32 (0.78, 2.23)
Preterm birth		20		0.74 (0.47, 1.16)		93		1.02 (0.82, 1.27)		113		0.95 (0.79, 1.16)
Very preterm birth		1		0.22 (0.03, 1.58)		20		1.37 (0.87, 2.14)		21		1.09 (0.70, 1.69)
SGA		35		1.04 (0.74, 1.47)		117		1.23 (1.03, 1.48)*		152		1.19 (1.01, 1.39)*
Term LBW		4		0.60 (0.23, 1.60)		37		1.68 (1.20, 2.34)**		41		1.42 (1.04, 1.94)*
**a**Models were adjusted for mother’s age, education, race, and number of previous live births; infant’s sex; and adequate prenatal care (Kessner index). *p < 0.05; **p < 0.01.												

**Table 3:** Adjusted RRs*^a^* (95% CIs) for birth defects in the in the PCE, TCE, and combined study areas, Village of Endicott, New York (USA), 1983–2000.

PCE area			TCE area			Combined study area		
Birth defect group*b*	*n*	RR (95% CI)	*n*	RR (95% CI)	*n*	RR (95% CI)
All reportable birth defects		17		1.24 (0.75, 2.05)		44		1.07 (0.78, 1.47)		61		1.11 (0.85, 1.45)
Surveillance birth defects		10		1.44 (0.72, 2.88)		25		1.43 (0.96, 2.14)		35		1.43 (1.01, 2.03)*
All cardiac defects		5		1.42 (0.46, 4.39)		15		2.15 (1.27, 3.62)**		20		1.97 (1.22, 3.16)**
Major cardiac defects		2		2.91 (0.73, 11.65)		6		2.40 (1.00, 5.77)*		8		2.53 (1.21, 5.31)*
Conotruncal defects		1		4.91 (0.69, 34.90)		3		4.91 (1.58, 15.24)**		4		4.92 (1.84, 13.11)**
**a**Models were adjusted for the mother’s age, education, race, and number of previous live births; infant’s sex; and adequate prenatal care (Kessner index). **b**There were no births in the study areas with NTDs, orofacial clefts, or choanal atresia; therefore, these outcomes are not shown. *p < 0.05; **p < 0.01.												

**Table 4:** Adjusted*^a^* RRs (95% CIs) for covariates included in the Poisson regression models of selected birth outcomes.

Birth weight, growth restriction outcomes (1978–2002 births)					Birth defect category (1983–2000 births)				
Covariate	LBW	SGA	Term LBW	Surveillance birth defects	Major cardiac defects	Conotruncal heart defects
Age (years)												
10–18 vs. 19–34		0.90 (0.88, 0.92)		0.85 (0.83, 0.86)		0.79 (0.77, 0.82)		0.97 (0.93, 1.01)		0.96 (0.86, 1.08)		0.93 (0.74, 1.15)
≥ 35 vs. 19–34		1.34 (1.32, 1.37)		1.07 (1.06, 1.08)		1.24 (1.21, 1.27)		1.14 (1.11, 1.17)		1.12 (1.04, 1.20)		1.17 (1.02, 1.33)
Education (years)												
12–15 vs. 0–11		0.71 (0.70, 0.72)		0.68 (0.67, 0.69)		0.64 (0.63, 0.65)		0.89 (0.87, 0.92)		0.83 (0.77, 0.89)		0.82 (0.72, 0.94)
≥ 16 vs. 0–11		0.51 (0.50, 0.51)		0.51 (0.50, 0.52)		0.42 (0.41, 0.44)		0.79 (0.76, 0.81)		0.68 (0.63, 0.74)		0.66 (0.56, 0.78)
Nonwhite vs. white		1.97 (1.94, 1.99)		1.66 (1.65, 1.68)		1.81 (1.77, 1.84)		0.88 (0.85, 0.90)		1.31 (1.23, 1.40)		0.87 (0.76, 1.00)
Male vs. female		0.85 (0.84, 0.85)		0.99 (0.98, 0.99)		0.66 (0.65, 0.67)		1.69 (1.66, 1.72)		1.19 (1.14, 1.25)		1.47 (1.35, 1.61)
Previous live births,												
1 vs. 0		0.68 (0.67, 0.69)		0.69 (0.69, 0.70)		0.67 (0.66, 0.69)		0.92 (0.91, 0.94)		1.02 (0.97, 1.08)		0.96 (0.86, 1.07)
≥ 2 vs. 0		0.69 (0.68, 0.70)		0.66 (0.66, 0.67)		0.69 (0.67, 0.70)		0.92 (0.90, 0.94)		1.11 (1.04, 1.18)		1.04 (0.93, 1.17)
Adequate prenatal care*b*		0.69 (0.68, 0.69)		0.78 (0.78, 0.79)		0.69 (0.68, 0.70)		1.01 (0.99, 1.03)		0.91 (0.87, 0.96)		0.95 (0.86, 1.04)
**a**The Poisson regression models for each outcome include residence in the combined study area versus the rest of New York State (excluding New York City) and all covariates in the table. **b**Adequate prenatal care is based on the Kessner index (Kessner et al. 1973).												

Between 1983 and 2000, 61 children born in the combined study area (17 in the PCE area, 44 in the TCE area) were diagnosed with at least one reportable birth defect (Table 3). There were no reported cases of NTDs, orofacial clefts, or choanal atresia in the TCE or PCE areas over the 18-year study period. All reportable, surveillance, and cardiac birth defect groupings showed elevated RRs for both areas combined (Table 3). Surveillance birth defects were increased in the PCE (*n* = 10) and TCE areas (*n* = 25), with a statistically significant elevation for the combined study area (RR = 1.43; 95% CI: 1.01, 2.03). Cardiac birth defects were increased in both areas, with RRs increasing from the most general grouping (all cardiac defects, 5 cases in the PCE area, and 15 in the TCE area) to most specific (conotruncal defects, 1 case in the PCE area, and 3 in the TCE area). In the TCE area, all cardiac defects were significantly elevated (RR = 2.15; 95% CI: 1.27, 3.62), whereas the major cardiac defects (*n* = 6) were of borderline statistical significance (RR = 2.40; 95% CI: 1.00, 5.77). In the combined study area, similar significant elevations were seen for all three groupings of cardiac defects. The highest RRs were for conotruncal defects and were statistically significant in the TCE area (RR = 4.91; 95% CI: 1.58, 15.24) and the combined study area (RR = 4.92; 95% CI: 1.84, 13.11). Although statistically significant, these RRs were based on three and four exposed cases, respectively.

## Discussion

Results of this study indicate that maternal residence in the TCE area, but not the PCE area, was associated with LBW, term LBW, and SGA. It is possible that lower levels of PCE and other VOCs in the PCE area, compared with TCE, were not high enough to observe an effect on birth weight and gestational growth. In addition, the small number of births in the PCE area made it difficult to estimate associations with a reasonable degree of precision. The increase in LBW births in the TCE area does not appear to be strongly related to shortened gestation. The fact that SGA and term LBW were both elevated suggests that the increase in LBW is related to *in utero* growth restriction rather than preterm birth.

Several studies of adverse birth outcomes in communities exposed to TCE and PCE have evaluated LBW and term LBW. In a cross-sectional study of 80,938 births in northern New Jersey, Bove et al. (1995) reported an association between levels of TCE in drinking water > 10 ppb and term LBW [odds ratio (OR) = 1.23; 50% CI: 1.09, 1.39]. In Tucson, Arizona, where part of the water distribution system was contaminated with TCE, Rodenbeck et al. (2000) studied 1,099 births to mothers living an area with TCE levels from 5 to 107 ppb and found an elevated but not significant risk of very LBW (OR = 3.3; 95% CI: 0.5, 20.6). Our results provide consistent evidence of associations between TCE and term LBW (RR = 1.68; 95% CI: 1.20, 2.34), as well as LBW (RR = 1.36; 95% CI: 1.07, 1.73).

Two studies found associations between SGA and TCE, consistent with our finding for the TCE area in Endicott. At Camp LeJeune, North Carolina, two water systems at the U.S. Marine Corps Base were contaminated with TCE (900–1,400 ppb) (ATSDR 1998). Researchers evaluated 31 births with “long-term” TCE exposure and 141 births with “short-term” TCE exposure and reported an association between SGA among male infants and “long-term” TCE exposure (OR = 3.9; 90% CI: 1.1, 11.9). In Woburn, Massachusetts, a retrospective cohort study found that the prevalence of SGA among 2,211 births was significantly elevated among mothers who had been exposed to drinking water contaminated with TCE (267 ppb) and PCE (21 ppb) during the third trimester of pregnancy (MDPH, CDC, and MHRI 1996).

Similar to our study, Aschengrau et al. (2008) found no association between PCE exposure and LBW or SGA. The retrospective cohort study evaluated 2,125 births in Cape Cod, Massachusetts, where PCE in drinking water reached levels as high as 7,750 µg/L. Sonnenfeld et al. (2001) reported only weak associations between PCE exposures (215 ppb) and the risk of SGA among 11,798 births to mothers at Camp LeJeune.

Surveillance birth defects and cardiac birth defects were elevated in both the PCE and TCE areas. Although similar in magnitude, the elevations were statistically significant only in the more populated TCE area. RRs for all cardiac defects and major cardiac defects for the PCE and TCE areas suggest an approximate doubling in the prevalence of these defects. When we limited the analysis to the more etiologically homogeneous group of conotruncal defects, the RRs suggest nearly five times greater prevalence of these defects in both the PCE and TCE areas. However, estimates were based on only four exposed cases, and CIs were wide. Animal studies suggest that TCE and its metabolites are specific cardiac teratogens (Dawson et al. 1990; Johnson et al. 2003); however, the mechanism of action of TCE (or PCE) on the developing heart is not well understood, and mechanisms may differ for individual defects.

Several epidemiological studies have reported an excess prevalence of cardiac defects in communities exposed to TCE-contaminated drinking water. Goldberg et al. (1990) conducted a case–control study in Tucson, where the drinking water was contaminated with 6–239 ppb of TCE. The prevalence of major cardiac defects in the exposed area was 2.5 times higher than in a nonexposed area, consistent with our present findings. In New Jersey, Bove et al. (2002) found associations between major cardiac defects and exposure to TCE levels > 10 ppb (OR = 1.24; 90% CI: 0.5, 2.9) and PCE levels > 5 ppb (OR = 1.16; 90% CI: 0.6, 2.1). In the only study to look at ambient air exposure to TCE, Yauck et al. (2004) reported an increase in heart defects among mothers ≥ 38 years of age living within 1.33 miles of a TCE-emitting site (OR = 3.2; 95% CI: 1.2, 8.7).

Although most published studies involved communities exposed to contaminated drinking water, the route of exposure in Endicott was inhalation from SVI. The different exposure pathways create a challenge for comparing exposure levels across studies. Inhalation exposures from contaminated water, during showering, for example, may result in similar levels of exposures as from ingestion. Haddad et al. (2006) estimated that a TCE concentration of 100 µg/L in drinking water results in a TCE concentration in shower air of 1,220 µg/m^3^ and in indoor air of 5.6 µg/m^3^. Based on these concentrations, applied to a “standard” adult male, their model resulted in a total daily absorbed TCE dose of 292.0 µg/day, of which 51% was from ingestion, 14% from indoor air inhalation, 22% from shower inhalation, and 12% from shower dermal absorption. Neither TCE nor PCE has ever been detected in public water in Endicott at levels exceeding federal or New York State standards of 5 µg/L. (ATSDR 1994, 2006). Thus, although the Endicott exposures did not include ingestion, the Endicott and comparison studies all include inhalation exposures.

*Strengths and limitations.* An important strength of our study was the use of available environmental and health outcome data. This approach allowed the study to be completed relatively quickly and inexpensively. Another strength is the use of statewide, individual-level birth data from the CMR and New York State Vital Statistics records. These data are gathered consistently for the entire state, are managed in centralized databases, and provide population-based data for births. Individual-level information on birth certificates enabled analytic control for other risk factors, including the sex of the baby; maternal age, race, and education; parity; and adequacy of prenatal care. In addition, the use of a large population (New York State excluding New York City) as a comparison population provides sufficient power to detect small associations between exposure and outcome.

A major limitation of this study, and most of the studies we cite, is that exposure at the individual level could not be measured and is largely unknown. The exposure assessment for the present study was based on soil vapor and indoor air sampling, which were used to determine the exposure areas. Although the study looked at births as far back as 25 years, indoor air sampling data was done between 2001 and 2004. However, not every house in the area was sampled, levels varied from house to house, and the amount of time spent in the house is unknown. In addition, some of the mothers may also have been employed by manufacturers in Endicott that used VOCs. If there were other VOC exposures in the community, the risks of adverse health outcomes associated with SVI in the residence would have been overestimated.

Exposure misclassification could have occurred from relying on the mother’s address on the birth certificate. Studies have estimated that 22–32% of women may move between the time of conception and delivery (Miller et al. 2010; Shaw and Malcoe 1992). The studies also report that similar numbers of cases and controls moved between the date of conception and the date of birth. Thus, any exposure misclassification that may have occurred would be expected to be nondifferential and would result in moving the RR toward the null. In addition, because of the overall decrease in the Endicott study area population, we believe that it is more likely that women moved out of the study area than moved in. This migration would potentially decrease the number of births and adverse birth outcomes in the study area, but the effect on the comparison population would be negligible.

Another limitation of the study was the small number of births in the study area and, consequently, a small number of cases for rarer outcomes. This was especially problematic in the PCE area, where nearly half of the outcomes had < 10 cases. However, even the larger TCE area had < 10 cases of some of the cardiac defect subcategories. This led to wide CIs around the RRs for some outcomes, and therefore, the results should be interpreted with caution.

The inability to adequately control for smoking is also a limitation for certain outcomes evaluated in this study. Cigarette smoking is the most important risk factor for fetal growth restriction (Kramer 1987). The Endicott area is a working class neighborhood, and analysis of the last 5 years of the study period showed a much higher prevalence of smoking among women during pregnancy there than in the rest of the state. In the subanalysis in which we controlled for maternal smoking during these years, the RRs for LBW, SGA, and term LBW all were lower, indicating that smoking was a confounder. If smoking patterns were similar for the full 25-year period, then RRs not adjusted for smoking may be overestimates. This suggests that smoking among women in the Endicott area may explain some of the elevations for LBW and SGA.

Another limitation is that the population of the study area is of lower socioeconomic status (SES) than the general comparison area population. Several of the outcomes are associated with lower SES, specifically LBW, term LBW, and SGA. We included mother’s education in the model to attempt to control for SES differences. Education was inversely associated with all of the birth outcomes. We included adequacy of prenatal care in the model because access to health care may be one of the pathways through which SES affects these birth outcomes. Although we used individual-level information for these important risk factors, the statistical modeling was not able to completely control for factors related to SES that are substantially different in the exposed versus the comparison population. An additional concern was that inclusion of race in the models, given its correlation with SES, could artificially inflate the exposure RRs for the predominantly white exposed population. Analyses that excluded race as a variable did show some slightly reduced RRs but did not affect the study’s overall results (data not shown). It is possible, however, that the models do not fully control for SES differences. Residual confounding by SES would lead to inflated estimates of risk associated with living in the Endicott study areas.

The study’s strongest finding is for an association of TCE and PCE exposures with cardiac birth defects, although some subgroups were based on a small number of cases. We found that the effects of education and prenatal care levels were greater on LBW and growth restriction outcomes than on birth defects. This is consistent with the interpretation that the birth defect findings are less likely to be artifacts of confounding by SES than are LBW and growth restriction. If the models controlled adequately for SES differences, as well as other biases, then living in the Endicott area is associated with increases for both types of adverse birth outcomes. The findings from the analyses that controlled for smoking were consistent with this interpretation; it showed a statistically significant elevation for term LBW, suggesting that smoking among women in the Endicott (a behavior associated with lower SES) could not fully account for the elevation of term LBW.

## Conclusion

This study is one of the first to examine a community with TCE and/or PCE exposure primarily through SVI. SVI is often discovered long after groundwater contamination begins, which makes historic exposure levels difficult to assess. This study adds to the larger body of evidence linking TCE, and possibly PCE, to adverse birth outcomes. We observed increased LBW, term LBW, and SGA among mothers living in the TCE area but not in the PCE area. We observed an increased prevalence of cardiac defects and, specifically, for conotruncal cardiac defects in the TCE and PCE areas; however, the latter result was based on just four cases. Nonetheless, this exploratory study suggests that additional research on exposures to VOCs and birth outcomes, particularly cardiac defects, is needed.
